# Impacts of NF1 Gene Mutations and Genetic Modifiers in Neurofibromatosis Type 1

**DOI:** 10.3389/fneur.2021.704639

**Published:** 2021-09-08

**Authors:** Wei Wang, Cheng-Jiang Wei, Xi-Wei Cui, Yue-Hua Li, Yi-Hui Gu, Bin Gu, Qing-Feng Li, Zhi-Chao Wang

**Affiliations:** Department of Plastic and Reconstructive Surgery, Shanghai Ninth People's Hospital, Shanghai Jiao Tong University School of Medicine, Shanghai, China

**Keywords:** neurofibromatosis type 1, genotype-phenotype correlation, modifier genes, NF1 gene, clinical variability

## Abstract

Neurofibromatosis type 1 (NF1) is a tumor predisposition genetic disorder that directly affects more than 1 in 3,000 individuals worldwide. It results from mutations of the NF1 gene and shows almost complete penetrance. NF1 patients show high phenotypic variabilities, including cafe-au-lait macules, freckling, or other neoplastic or non-neoplastic features. Understanding the underlying mechanisms of the diversities of clinical symptoms might contribute to the development of personalized healthcare for NF1 patients. Currently, studies have shown that the different types of mutations in the NF1 gene might correlate with this phenomenon. In addition, genetic modifiers are responsible for the different clinical features. In this review, we summarize different genetic mutations of the NF1 gene and related genetic modifiers. More importantly, we focus on the genotype–phenotype correlation. This review suggests a novel aspect to explain the underlying mechanisms of phenotypic heterogeneity of NF1 and provides suggestions for possible novel therapeutic targets to prevent or delay the onset and development of different manifestations of NF1.

## Introduction

**Figure 1:**
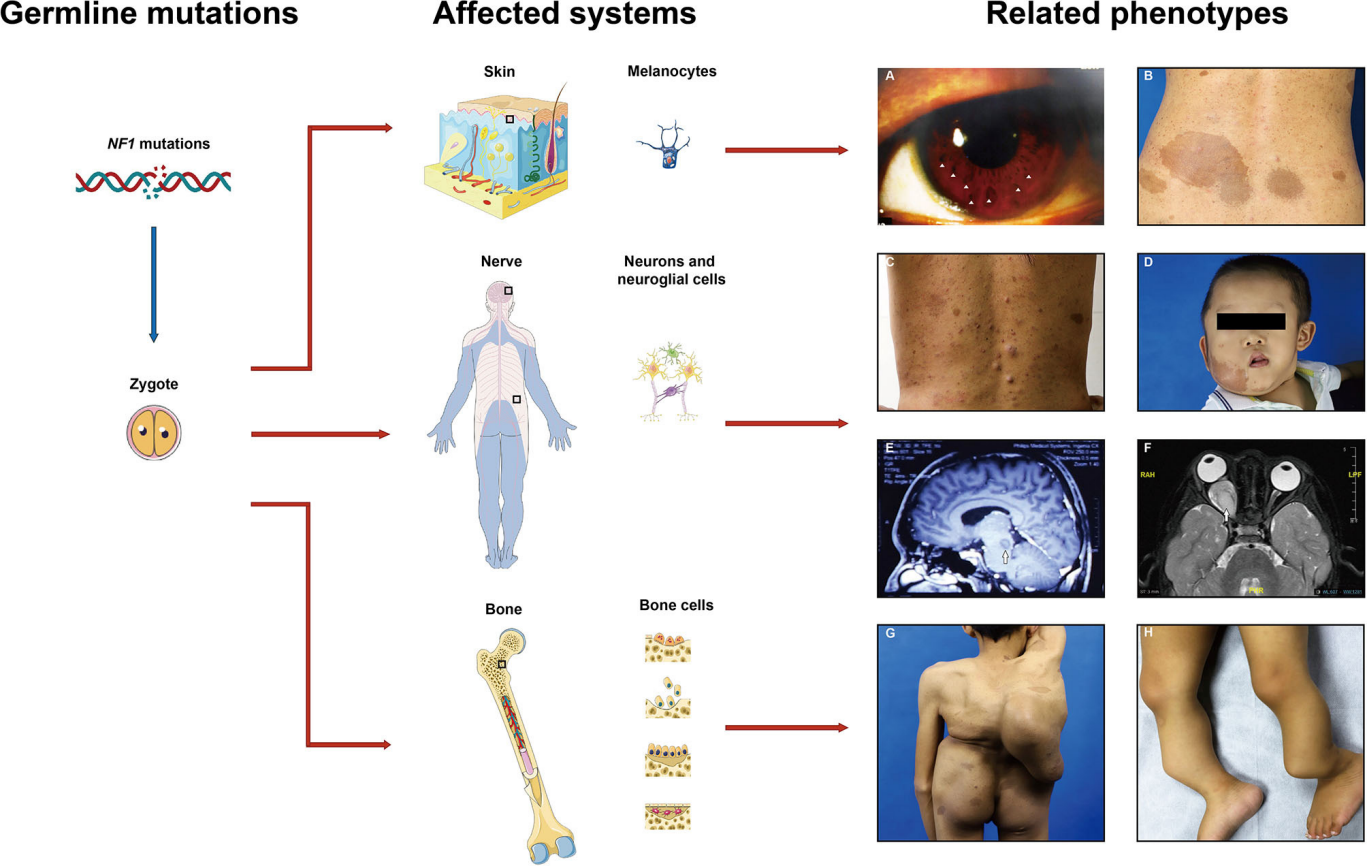
Common affected systems and related phenotypes in neurofibromatosis type 1 (NF1) patients. Diverse clinical features present due to the *NF1* gene mutations in different cell types including melanocytes, neurons, neuroglial cells, osteoblasts, etc. **(A)** Lisch nodules, **(B)** cafe-au-lait macules, **(C)** cutaneous neurofibromas, **(D)** plexiform neurofibromas, **(E,F)** gliomas, **(G)** scoliosis, **(H)** pseudoarthrosis. Images are used with patient permission.

Neurofibromatosis type 1 (NF1), a relatively common tumor predisposition genetic disorder, affects ~1:3,000–3,500 live births worldwide (1). NF1 patients present lifelong phenotypic variability with almost complete penetrance (Figure 1). Common clinical NF1-related manifestations based on the NIH diagnosis criteria include multiple cafe-au-lait macules (CALMs), Lisch nodules, cutaneous/dermal neurofibromas (CNFs), plexiform neurofibromas (PNFs), and osseous defects (2). Other distinctive features such as learning disability and childhood overgrowth are also reported (3). Neoplastic complications are also associated with NF1 and greatly affect life quality, including malignant peripheral nerve sheath tumor (MPNST), optic glioma, leukemia, and breast cancer (4–7). Clinical expressivity among NF1 patients is variable, unpredictable, and age-related. Exploration of the underlying mechanisms of these variabilities is important for further personalized healthcare and makes prognostic counseling easier (8).

Studies showed that both the *NF1* mutations and modifiers may correlate with the variations in clinical phenotype. Genotype–phenotype relationships provide an approach to understand the pathogenesis and development of NF1. On the one hand, NF1 is a genetic disorder resulting from mutations in the *NF1* gene. This gene, located on chromosome 17, band q11.2, is a large and complex gene, which carries more than 280 kb of genomic DNA, including 57 constitutive exons and other alternative spliced exons (9). The overall pathological *NF1* mutation rate is 92.1% (70/76) in NF1 individuals detected by next-generation sequencing (10). Till now, over 2,800 different *NF1* variants have been identified (11). Certain genotype–phenotype correlations have been identified, although most mutations are inactivating. That cannot simply answer the variable expressivity, as more severe manifestations can be observed in siblings even with the same germline mutation in NF1 families and vice versa (12). This indicates that modifier genes may play a vital role for the differences in clinical manifestation besides genotype–phenotype correlation (11, 13). Theoretically, modifiers themselves do not have clinically pathogenic features, but they affect target genes which related to a certain genetic disease and modulate phenotypic manifestations (14–17). Identification of *NF1* genotype–phenotype correlations and genetic modifiers is pivotal to understanding the underlying molecular mechanisms of pathogenesis, progression, and expressive variety in NF1.

This review outlines the current studies of *NF1* genetic heterogeneity and modifier genes in NF1. The differences in the mutations of *NF1* gene and related modifiers are described according to variable phenotypes (summarized in Table 1). Understanding their correlation will help for earlier diagnosis, probable target-driven therapies, and prognostic prediction of NF1 patients.

**Table 1:** A list of mutations of *NF1* gene and genetic modifiers that related to different NF1 phenotypes involved in this article.

**Phenotypes**	***NF1* gene mutations**	**Characteristics**	**References**
Cafe-au-lait macules			
*NF1* gene mutations	992p.Met992del	Extinct CALMs and absence of neurofibroma	(18)
	p.Arg1809 substitution	Extinct CALMs and absence of neurofibroma	(19)
	p.Arg1038Gly	Extinct CALMs and absence of neurofibroma	(20)
Lisch nodules			
*NF1* gene mutations	Frameshift	–	(20–22)
Cutaneous neurofibromas			
*NF1* gene mutations	p.Met992del, p.Arg1809 substitution and p.Arg1038Gly	Mild phenotype with CALMs and absence of other types of neurofibromas	(18–20)
	Microdeletions	Higher number of CNFs	(23–25)
Plexiform neurofibromas			
*NF1* gene mutations Modifier genes Cognitive disorders	Microdeletions Runx1 ANRIL SUZ12 ATM	High PNFs burden (>3,000 ml) Tumor-suppressor gene or oncogene PNF number and malignant progression PNF formation and progression Initiate PNF formation and increase tumor number	(26) (27) (28) (29) (15)
*NF1* gene mutations	Exon 23a	Hippocampal learning disability, cognitive impairment, and others	(30, 31)
	Exon 9a	Synaptic plasticity and learning behavior	(32)
Modifier genes	Nmdar I	Hippocampal mediated learning, memory, and spatial learning	(33)
	Rabs, synaptotagmins, CaMKII, and CREB1	Synaptic plasticity	(34)
Osseous lesions			
Modifier genes Childhood overgrowth	ATF4 serum 25(OH) vitamin D/VDR	Enhance skeletal development Regulation of calcium homeostasis and bone mass	(35) (36, 37)
*NF1* gene mutations	NF1 microdeletion in 17q11.2	Taller and heavier in young children	(3)
Cardiovascular malformation *NF1* gene mutations	RNF135/*NF1*-REPa to REPb deletion microdeletion	Overgrowth syndrome Cardiac anomalies	(38) (39)
Modifier genes	ADAP2	Congenital valve defects	(40)
	CENTA2 and JJAZ1	Cardiovascular malformation	(39)
Malignant peripheral nerve sheath tumor			
*NF1* gene mutations Modifier genes NF1-associated optic glioma *NF1* gene mutations Modifier genes	844–848 Missense mutations TP53/p53, CDKN2A/p16 and PTEN MDM2 p.R681X Enrichment in the 5′ region PTEN ADCY8	Earlier PNF onset and increased risk for MPNSTs Malignant development Decreased survival rate Increase tumor onset and optic nerve volume Potential link to higher incidence of optic glioma development Increased astrocyte proliferation and optic glioma growth Sexually dimorphic growth in female	(41) (42, 43) (44) (45, 46) (47) (5) (48) (49)
Modifier genes of other non-neurofibroma tumors in NF1	BRCA1/2 MLH1 ASXL1 and p19ARF	Early-onset breast cancer Early-onset leukemia Development of leukemia	(50) (51) (52, 53)

## Mutations of *NF1* or Modifier Genes Related To Different NF1 Phenotypes

### Clinical NF1-Related Manifestations

#### Pigmentary Features

CALMs are regarded as the most common pigmentary feature of NF1 caused by biallelic loss of NF1 in melanocytes (54). They can be detected in 2.7% of newborns and 28% of young children (55). These macules also are the earliest manifestation of NF1, with great diagnostic importance in younger children. They may have a higher risk for NF1, which could be warranted later by other features (55).

Several studies have shown that certain NF1 mutations give rise to CALMs and freckling only phenotypes without any visible neurofibromas. A study of 21 unrelated NF1 probands and 26 affected relatives shows that individuals with a 3-bp loss of a single amino-acid deletion at codon 992 (p.Met992del), 0.9% frequency in NF1 mutation individuals, present a mild clinical phenotype with CALMs and absence of neurofibroma manifestations (18, 19). A 3-bp in-frame deletion (c.2970-2972 delAAT) in exon 17 of the NF1 gene results in the loss of one of two adjacent methionines (codon 991 or 992), which may affect the expression of the highly conserved region of neurofibromin (55). Further research on 135 individuals from 103 unrelated families with p.Met992del also confirms this result (19). Moreover, researchers identify a certain ratio of other complications including 4.8% non-optic brain tumors and 38.8% cognitive/learning disabilities in NF1 patients with this genetic change (19). Taken together, the presence of >5 CALMs and armpit freckling can be observed in a dramatic 166/182 (91.2%) and 103/171 (60.2%) cases carrying the NF1 p.Met992del pathogenic variant, according to the combined data of these two studies (18, 19). Even prolonging the observation period for over 9 years, neurofibroma manifestations such as CNFs or PNFs do not occur (19). Another study has identified that p.Arg1809 substitution, a c.5425C4T missense variant within exon 38, exhibits clinical similarities to the cohort data from the p.Met992del single amino acid deletion in 0.7% of total 786 NF1 patients (56). This change rearranges the intradomain structure of the plekstrin homology-like domain of GTPase and confers a high predisposition to pigmentary signs without other common NF1 neurofibroma-related manifestations, such as CNFs or PNFs (56). Besides the above two, the missense variant c.3112A>G, p.Arg1038Gly of *NF1* gene, tested in seven patients from two unrelated families, is also associated with CALMs without other types of neurofibromas (57).

These genotype–phenotype correlations in NF1 have presented possible pigment-predominant tumor-free phenotypes. It is noteworthy due to the fact that CNFs are the commonest feature of NF1. More importantly, current diagnosis criteria are based on clinical symptoms, requiring multiple manifestations such as CALMs and neurofibroma for diagnosis. It is hard to fulfill the diagnosis criteria when these special *NF1* mutations are present. These findings indicate that there should be future consideration of these special NF1 patients with isolated CALMs in clinical diagnosis, especially for children younger than 29 months with 6 or more CALMs (55).

Lisch nodules are another typical pigment feature in NF1 diagnosis and the most common ocular manifestation in NF1. They are benign tumors located on the iris surface and present to be well-defined, gelatinous pigments (58). Lisch nodule burden may be correlated with choroidal abnormalities in patients with NF1 (59). In a genetic study of 84 NF1 patients, including 25 siblings, 26 children, 30 parents, and 3 grandparents, the Lisch nodule phenotype presents to be more common (22.6% vs. 9.1%) carrying frameshifting mutations (21). This result is consistent with previous observations by Sabbagh et al. (22) and Castle et al. (20).

#### Cutaneous Neurofibromas (CNFs)

CNFs, also called dermal neurofibromas, exclusively grow within the cutaneous dermis layer and form hundreds or thousands of small tumors. They show no propensity to malignancy (60, 61). More than 99% of adult NF1 patients present CNFs (62). They frequently develop and increase in number from early adolescence until late adulthood (57).

Patients with three *NF1* gene mutations, p.Met992del, p.Arg1809 substitution, and p.Arg1038Gly, exhibit a mild phenotype of NF1, which are associated with a lack of CNF manifestation. In an estimated 5–10% of all NF1 patients, microdeletions encompassing the entire *NF1* gene and flanking regions at 17q11.2 are responsible for more severe features. *NF1* microdeletions share the same definition with non-mosaic large *NF1* deletions (23, 24). Patients with NF1 microdeletions are prone to a remarkably higher robust number of CNFs. In a study of Plotkin et al., 50% (10/20) of adult patients show a very high burden of CNFs, with over 1,000 in total number (25). However, although CNFs are a relatively common feature for NF1 patients, the genetic changes specific to this phenotype are not fully understood. Identification of mutations and genotype–phenotype correlation in NF1 may better explain the tumorigenesis; physical variabilities in tumor type, density, and size; differences in growth speed of the proliferative process; or the co-occurrence of other phenotypes in CNFs.

#### Plexiform Neurofibromas, PNFs

Twenty to fifty percent of NF1 patients present with PNFs (63). In contrast to CNFs, PNFs have a different developmental origin and grow deeper, along internal nerve plexus cranial or large peripheral nerve sheaths. PNF is congenital and grows slowly, except for the periods of early childhood and pregnancy (64). The size and location of the tumor range determine the patients' main complaints, which include facial defects, nearby-organ compression, deformities, or invasion and may further lead to physical pain and functional damage (65, 66). PNFs can turn into MPNSTs, which occupied around 8–13% NF1 patients in total (67).

Extremely high PNF burden (>3,000 ml) exists significantly more frequently among NF1 patients with non-mosaic large *NF1* deletions (13% vs. 1% in patients without large *NF1* deletions) (25, 26). Patients with higher tumor burden will in turn have a relatively higher risk to develop MPNSTs and other severe features than non-deletion NF1 patients (25, 68, 69). As for modifiers, ANRIL is especially relevant to the PNF number (28). It affects the expression of CDKN2A/ARF and CDKN2B tumor-suppressor genes, which further interrupts the cell cycle and apoptosis in PNF and other cancers (28, 70–72). A study from Li et al. underlines the important role of Runx1, functioning paradoxically either as a tumor-suppressor gene or as a dominant oncogene in NF1 neurofibroma initiation. This overexpression of Runx1 is proved in Schwann cell progenitors and neurofibromas in the classic *Nf* 1 fl/fl; DhhCre mouse model. In this model, Li and colleagues confirmed that by inhibiting the expression of Runx1, the sphere number of neurofibromas reduced. The decrement of stem-like progenitor cells and cell growth can explain this change (27). Other genes (RNF43), or molecular pathways (Wnt/β-catenin pathway), correlate with the tumorigenesis of PNFs and can also act as candidate genes (73, 74).

Their propensity for malignancy has been studied over the years. The expression status of ANRIL may further prompt the development of PNFs into premalignant tumors such as atypical neurofibromas (ANF) or atypical neurofibromatous neoplasms of uncertain biologic potential (ANNUBP) (75). In another cohort study, Pasmant et al. point out that SUZ12, a gene combining and inactivating ANRIL, is a modifier gene of PNF formation and progression (29). Recently, ATM is proven to be another modifier gene of NF1. It is a DNA repair-related gene, with an increased neurofibroma tumor load or malignant transformation, when overexpressed under the biallelic NF1 mutation background (15). Its heterozygosity initiates PNF formation and increases the tumor number in a *Nf1-*deficient mouse model by stimulating SCP self-renewal and promotion of tumorigenesis (15).

Taken together, different types of *NF1* gene mutations and modifiers play an essential role in PNF tumorigenesis and tumor development. However, larger cohort individuals are still needed for verification and clarification.

#### Learning Disability and Other Cognitive Disorders

Learning disabilities are one of the most frequent cognitive disorders in NF1. Up to 75% of children with NF1 have learning problems, who suffer from academic deficiency, especially in mathematics and reading (76, 77). Other typical cognitive disorders include motor skill impairment, attention-deficit/hyperactivity disorder, and intellectual disability (78).

It is reported that significantly reduced expression levels of neurofibromin isoform I mRNA are correlated with a severe phenotype of NF1 features, including learning disabilities/cognitive deficit, optic gliomas, and/or neoplasm/cerebrovascular disease, by analyzing the levels of two neurofibromin isoforms in circulating leukocytes of a cohort study (31). Moreover, this finding indicates the potential role of NF1 transcript processing in modulating NF1 phenotypic severity. GEM and other animal models can also display irreplaceable roles in genetic and pharmacologic studies and provide insights into trials with high and repeatable fidelity (79). Nf1-mutant mice with certain genetic changes unveiled abnormalities and underlying mechanisms in learning and memory impairments and other behavioral deficits. Alternative splicing of exon 23a inhibits Ras-GAP functions, which is crucial for brain development and cognitive functions (80, 81). The lack of exon 23a in neurons specifically results in defective associative fear learning and spatial memory in mouse models (30, 31). Like exon 23a, the exon 9a of *Nf1* also has a critical role in synaptic plasticity and learning behavior in the central nervous system (32). The loss of only *Nf1*-exon 9a in a transgenic mouse model leads to spatial learning and hippocampal plasticity deficits (32). Heterozygous null mutation of Nmdar I, a glutamate-gated ion channel, plays a critical role in hippocampal-mediated learning and memory and exacerbates the spatial learning phenotype of Nf1 +/– mutant mice (33). This genetic modifier alters the expression of NF1-related learning impairment phenotypes (33). Other genes that may play critical roles in synaptic plasticity, including Rabs, synaptotagmins, Calcium/calmodulin-dependent protein kinase II (CaMKII), cAMP-responsive element-binding protein 1 (CREB1), oligodendrocyte-myelin glycoprotein (OMG), and Cyclin-dependent kinase 5 regulatory subunit 1 (CDK5R1), could be regarded as candidate modifier genes of cognitive disorders (34, 39).

#### Osseous Lesions

Orthopedic manifestations, including scoliosis, osteoporosis, skull defects, tibial dysplasia, and pseudarthrosis, as well as reduction in musculature strength, have been reported to affect ~50% of patients with NF1 (82, 83). Some features may show sex predominance and are associated with other clinical NF1 phenotypes (84–86). Decreased neurofibromin on bone turnover, calcium homeostasis, and pubertal development may contribute to low bone mineralization and may further cause bony lesions (87). Identifying novel pathogenic variants in the NF1 gene helps to understand the genetic background, phenotype inclination, disease process, and therapeutic targets. This process benefits from both NF1 families and NF1-related animal models. In a recent large cohort study with 365 NF1 subjects included, whole-gene deletions and frameshift variants are found correlated with skeletal abnormalities, including scoliosis and sphenoid bone dysplasia (88). From another 10 unrelated Chinese families who affected NF1 with main complaint of osseous lesions, five novel pathogenic variants including one missense variant and four frameshift variants are detected (89). As for animal models, the loss of Nf1, specifically in limb osteoprogenitor cells (Nf1 Col2 model; Nf1 Prx1 mice) or osteoblasts (Nf1 Ob2/2 model), leads to dysfunctional and malformed limbs with abnormal joint cartilages and bones (35, 90–92). These NF1-related osseous defects are linked to hyperactive TGF-β1, RAS/ERK, JNK, and mTORC1 signaling pathways and can potentially be useful adjunctive agents for orthopedic medicine (91, 93–95).

Taken together, mutations of NF1 genes play an essential role in NF1-related osseous lesions. Moreover, other studies reveal that several factors contribute to this symptom. Modifiers including ATF4, an osteoblast-enriched transcription factor, can enhance skeletal development by increasing amino acid import and collagen synthesis in NF1-deficient osteoblasts (35). Moreover, the concentrations of serum 25(OH) vitamin D and its receptor (VDR), both responsible for the regulation of calcium homeostasis and bone mass, decrease and significantly correlate with higher tumor incidence in NF1 patients (36, 37). These related factors may contribute to osseous lesions by affecting the expression of other genes, which merits further investigation.

#### Other Non-neoplastic Features Not Mentioned Above

Childhood overgrowth with bone age acceleration is an unusual phenotype of NF1 resulting from a large 1.4/1.2-Mb NF1 microdeletion in 17q11.2 (3). Young children with microdeletions grow taller and heavier than those without these deletions (69). In addition, both loss-of-function mutations of ring finger protein 135 (RNF135) and microdeletion of NF1-REPa to REPb including RNF135 contribute to an overgrowth syndrome including tall stature, macrocephaly, dysmorphic features, and variable additional features in NF1 patients (38).

NF1 patients also present cardiovascular malformations. ADAP2 is a reliable candidate gene for the occurrence of congenital valve defects by affecting heart development. It has a higher incidence in *NF1*-microdeleted patients (40). By establishing an ADAP2 loss-of-function zebrafish model, defects in heart jogging and looping as well as defective valve development in ADAP2 morpholino oligo-injected embryos are observed (40). Moreover, CENTA2 and JJAZ1 are considered to be two possible candidates for cardiovascular malformations by analyzing clinical and genetic data from 92 *NF1*-microdeleted patients (39). *NF1* microdeletions should be considered especially when NF1 patients sign with dysmorphisms, cardiac anomalies, and learning disability (39).

### NF1-Related Neoplastic Complications

#### Malignant Peripheral Nerve Sheath Tumor, MPNST

NF1 patients have an 8–13% lifetime risk of developing MPNST, and ~50% of MPNSTs occur due to malignant transformation from PNFs (67). Before the MPNST stage, PNFs can be further divided into ANFs and ANNUBPs by histology with multiple atypical features; this stage is a kind of premalignant tumor stage (75). MPNSTs significantly reduce life expectancy with no gender preference in NF1 patients (96). Meta-analyses from 1963 to 2012 indicated a worse outcome of MPNSTs in patients with NF1 syndromes compared with non-NF1 patients (97). Loss of NF1 is a necessary but not sufficient approach to the promotion of PNFs into MPNSTs. Therefore, identifying NF1 individuals predisposed to developing malignancy is of great importance.

Missense mutations affecting *NF1* codons 844–848 have accelerated PNF formation at an earlier age, increased lifetime risk for MPNSTs, skeletal abnormalities, and ~0.8% prevalence in unrelated NF1 patients (41). Researchers made this conclusion by constituting a cohort of 162 NF1 individuals from 129 unrelated families to evaluate the prognosis of patients with these missense mutations (41). Seventy-five percent of NF1 patients with these missense mutations are observed with severe phenotypes. Patients increase PNF incidence from 15–30 to 39% over an observation period of more than 9 years, and the rate of malignant development to MPNSTs has reached 5% (7/139) (41).

As for modifiers, inactivation of the TP53/p53 gene, deletion of the CDKN2A/p16 gene, and loss of PTEN are all important in the progression from low-grade neurofibroma (atypical or low-grade malignancy) to MPNST under the effect of biallelic inactivation of *NF1* (42, 43, 98). In MPNST, complete TP53 mutations are present in up to 8.2–16.9% of patients, suggesting a poor prognostic phenotype (44). Consistent with TP53, amplification of MDM2 had a prevalence of approximately 5.5% (44). Studies have shown that with either type of these two aberrations, the 5-year disease-specific survival rate would significantly decrease in NF1 patients (44). The CDKN2A/p16 gene is also important in NF1. In a study of a single patient with three different kinds of neurofibromas at the same time, a neurofibroma, a low-grade MPNST, and a high-grade MPNST, homozygous loss of the CDKN2A/p16 gene is detected in the malignant component, especially during the progression or occurrence of high-grade MPNST (42). During the malignant process, the expression level of PTEN in MPNST reduces, whose degree is dependent on the tumor stages (43). Haploinsufficiency or complete loss of PTEN may significantly accelerate PNF development as well (43).

#### NF1-Associated Optic Glioma, OPG

NF1 optic pathway gliomas are commonly seen in ~15–20% of young children with NF1 (99). The majority of OPG patients suffer in childhood, younger than 7 years of age, and this lesion, localized within the optic pathway or brainstem, can result in unilateral proptosis, visual acuity loss, or field defects (100). It is unpredictable and requires routine surveillance.

An *Nf1* non-sense mutation in exon 13, c.2041C>T (p.R681X), causes truncation of neurofibromin in an *Nf1* ^+/−^ engineered mouse model and leads to the development of optic gliomas with increased optic nerve volumes (45, 46). Enrichment of mutations in the 5′ region of the *NF1* gene also suggests a higher incidence of optic glioma development (47). Moreover, heterozygous loss of the PTEN gene in the *Nf1* mutant mouse model increases *Nf1*-deficient astrocyte proliferation and optic glioma growth (101). In contrast, from the data of another larger cohort study, no direct studies prove the genotype–phenotype correlation between the clustering mutations in the 5′ region of the *NF1* gene and the presence of OPG in NF1 patients (102). Further studies are needed to verify their relationship.

Studies show that some specific mutations and clinical features are more prevalent in females. For this reason, sex is considered to be a major clinical modifier of neuronal dysfunction in NF1. Sexual dimorphism exists in the cAMP pathway and especially interrupts the PDE activity, which may influence the application and efficacy of specific inhibitors for NF1 patients. The polymorphism of cAMP synthetase, adenylate cyclase 8 (ADCY8), elevates the glioma risk in NF1 female patients, but reduces it in males (48, 103). This sex-specific manner may be due to the ADCY8-related sexually dimorphic growth that has opposing effects on Nf1^−/−^ astrocytes. By using the *Nf1*-deficient mouse model, it shows that females are more prone to OPG-associated visual decrement while males were solely prevalent in spatial learning deficits (49). All these data call for a more thorough consideration with matched patient sex, when developing drugs targeting NF1-associated optic glioma or brain tumor.

#### Other Non-neurofibroma Tumors in NF1

Besides neurofibromas and gliomas, there have also been some reports discussing the association between NF1 and other tumors, especially those with malignant characteristics, including breast cancer or leukemia. A recent epidemiologic survey focusing on the incidence of different kinds of cancer among 8,003 NF1 patients has identified an increased risk of many individual cancers (104). Four percent of NF1 patients are diagnosed with cancer. In particular, the rates of chronic myeloid leukemia and female breast are 6.7 and 2.3%, respectively, in this NF cohort (104).

Compared to healthy females, female NF1 patients have an estimated 2-fold increase in lifetime risk of breast cancer (18.0%) (7). For NF1 females <50 years old, the risk increases to 5-fold that of healthy females, accompanied with more advanced stage and higher mortality (105). This means that more aggressive features of breast cancer, such as higher tumor grade, negative estrogen receptor, HER2 amplification, and inferior overall survival, are evident among female patients with NF1 compared to the age-matched population (7, 106, 107). By using mouse models, scientists have confirmed the loss of *Nf1* and their link to breast cancer tumorigenesis (108). However, there are limited explanations about the mechanisms underlying this phenomenon. The co-occurrence of *NF1* and BRCA1/2 germline mutations may suggest a potential link to early-onset breast cancer in NF1 patients and calls for the study of more cases to obtain a convincing conclusion (50).

The association between leukemia and NF1 is controversial and needs to be further investigated. Early-onset leukemia may result from the presence of the homozygous MLH1 gene, a member of the DNA MMR genes with a concomitant *NF1* gene mutation (51). The loss of ASXL1 or p19ARF can accelerate the development of leukemia in the haploinsufficient *Nf1* mouse model, which may be due to the promoter methylation or activation of the MYC and MAPK pathway, respectively (52, 53).

Other kinds of non-neurofibroma tumors in NF1 patients, including pheochromocytoma, astrocytoma, and gastrointestinal stromal tumors, have also been reported in several studies (109–111). The c.1906G > C germline mutation in DNA mismatch repair genes that affects the expression of hMLH1, hMSH2, and hMSH6 may link to NF1 childhood malignancies (112).

**Figure 2:**
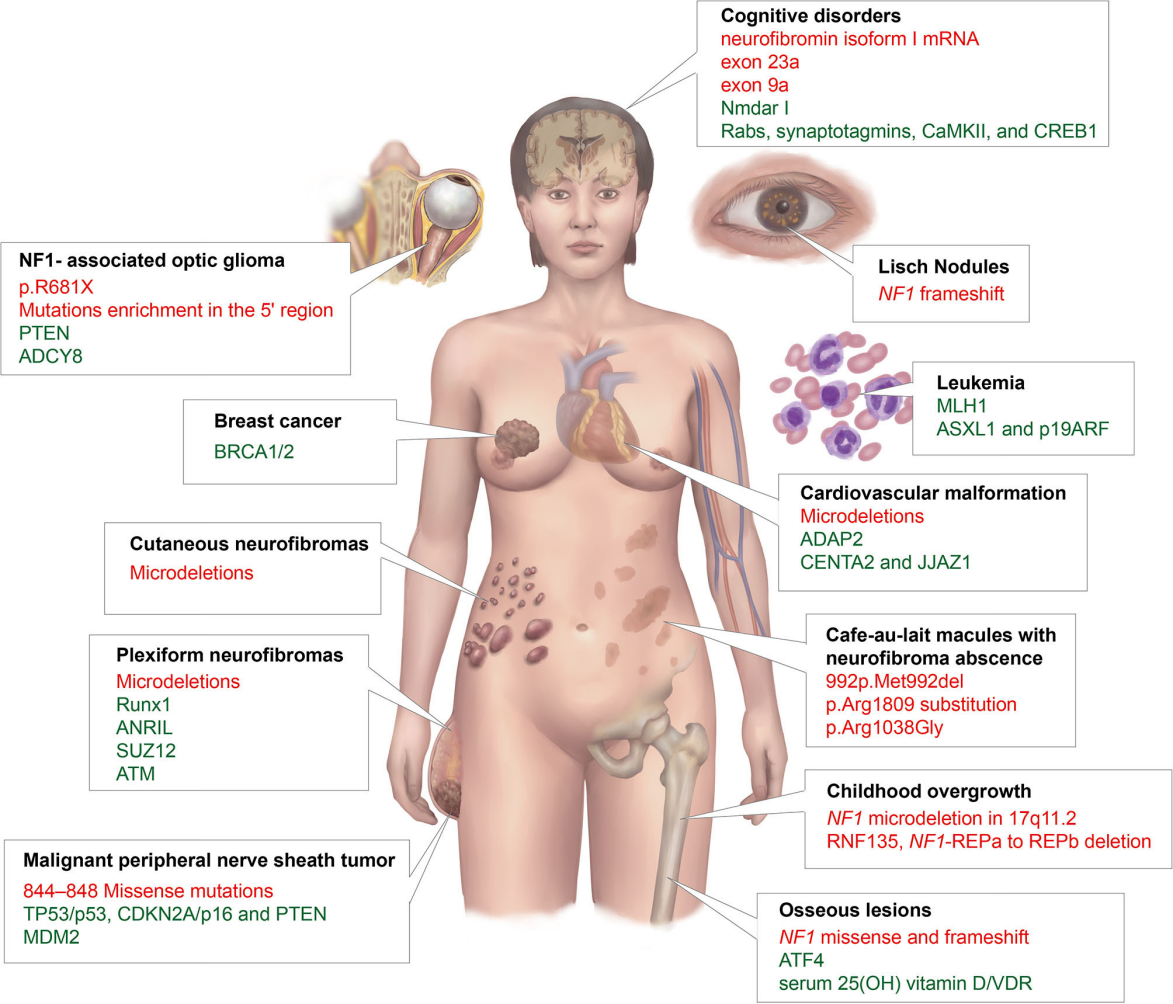
A summary of different NF1 phenotypes that are related to mutations of the *NF1* gene or genetic modifiers in this article. The black font represents phenotypes; red, *NF1* mutations; and green, genetic modifiers.

We summarized and displayed the different NF1 phenotypes related to mutations of the NF1 gene or genetic modifiers that mentioned in this review in Figure 2.

## Discussion

NF1 shows complete genetic penetrance but variable expressivity. Although the diagnostic criteria of NF1 are well-established, the varieties of clinical phenotypes of NF1 indicate possible challenges in the diagnosis of some special patients and treatments are considered only after clinical symptoms. For example, young children with NF1 may not have enough findings to make a clinical diagnosis, especially when no positive family history exists. In this circumstance, advanced techniques for blood analysis, such as next-generation sequencing, are recommended to evaluate the potential role and mutant frequency of different genes in NF1. Moreover, genetic counseling is needed to discuss the risk for severe phenotypes or even malignancy. For instance, patients with certain *NF1* mutants or suspected modifiers such as TP53 may be considered at higher risk for MPNST, which merits more careful personalized healthcare.

Other mechanisms that explain the variable phenotypes of NF1 are important, apart from NF1 gene heterogeneity and modifiers. Besides NF1-related neoplasms, they also affect sporadic or *de novo* tumors. These mechanisms included the following. (1) Epigenetic regulators. They affect the biochemical alterations in the development of NF1-linked tumors especially in malignant transformation process or tumor grades. For example, the epigenetic regulator polycomb repressive complex 2 (PRC2) plays an important role in malignant transformation to MPNST. The loss of PRC2 decreases dimethylation and trimethylation of H3K27me3 production and causes epigenetic changes *via* EED or SUZ12 mutation. By promoting hyperactive RAS signaling and reducing immune surveillance, the loss of PRC2 contributes to the MPNST tumorigenesis (113, 114). Due to the frequent inactivation of PRC2, its catalytic and independent component, the enhancer of zeste homolog 2 (EZH2), can be a potential therapy target in treating MPNST as this enhancer lacks mutation (115). Another example is glioma. It is reported that high-grade NF1 glioma exhibited frequent ATRX mutations, while a particular methylation subgroup of sporadic gliomas, the LGm6 subgroup, is enriched with ATRX mutations and assembles epigenetic profiles of NF1 glioma (116). Moreover, the epigenetic regulator can also provide a potential target when dealing with different phenotypes (117). (2) Mosaicism. It is an uncommon alteration of NF1 resulting from somatic mutations with mild and segmental presentation. Most children (65%) present with localized pigmentary only, while older patients are prone to present with neurofibromas (12, 118). Other presentations can also exist. Appropriate genetic counseling is needed as gonadal mosaicism can lead to complete NF1 manifestations in offspring (119). (3) Environment, such as hormone and vitamin D (12, 120). Collectively, these observations confirmed the impact of critical epigenetic, mosaic, and environmental elements on the pathogenesis of neurofibromas or other features.

For better understanding the genetic mechanisms that underlie the different NF1-associated features, clinical trials were applied to identify the genetic modifiers involved in the variability of the clinical expression of NF1 (Clinicaltrials.gov ID: NCT01650142), certain mutations, and phenotypes (Clinicaltrials.gov ID: NCT04212351) or evaluate the possibility of novel and genomically guided therapies (Table 2). There are several clinical trials targeted to mutate genes for NF1 patients. A completed phase II trial evaluated whether the selected drugs, sunitinib or everolimus, based on the defective gene would result in a better tumor response to patients with advanced neuroendocrine tumors, including NF1 (Clinicaltrials.gov ID: NCT02315625). Another trial studied the clinical activity of MEK1/2 inhibitors and dabrafenib combination to treat cancers especially PNFs harboring V600 mutation (Clinicaltrials.gov ID: NCT02124772). If the defective gene gets better results, it will be promising to develop gene-targeted drugs and treat NF1 patients with certain phenotypes. Another approach for application is to use vaccines that are made from a gene-modified virus in order to kill MPNST tumors that are either unresectable or recurrent with *NF1* mutation (Clinicaltrials.gov ID: NCT02700230). It may also help the body build a more effective immune environment to kill tumor cells. Researchers also designed clinical trials to study the effectiveness of a glutaminase inhibitor, CB-839 HCl, in treating patients with *NF1* aberrations, *NF1* mutant MPNSTs, or other aberrant solid tumors (Clinicaltrials.gov ID: NCT03872427). This inhibitor works by blocking glutamine activity needed for the growth of cells and may target the uncontrolled cell growth resulting from gene mutations.

**Table 2:** A list of clinical trials that identify the genetic modifiers, *NF1* mutations, phenotypes, or gene therapies of NF1.

**ClinicalTrials.gov ID**	**Study name**	**Recruitment status**	**Intervention**	**Phase**
NCT01650142	Modifying Genes in Neurofibromatosis 1	Unknown	–	–
NCT04212351	Frameshift Peptides of Children with NF1	Recruiting	Genetic: frameshift array blood sample test	–
NCT02315625	Study of Mutation-Targeted Therapy with Sunitinib or Everolimus in People With Advanced Low- or Intermediate-Grade Neuroendocrine Tumors of the Gastrointestinal Tract and Pancreas With or Without Cytoreductive Surgery	Completed	Sunitinib or everolimus	II
NCT02124772	Study to Investigate Safety, Pharmacokinetic (PK), Pharmacodynamic (PD) and Clinical Activity of Trametinib in Subjects with Cancer or Plexiform Neurofibromas and Trametinib in Combination With Dabrafenib in Subjects With Cancers Harboring V600 Mutations	Active, not recruiting	Trametinib or combination with dabrafenib	I/II
NCT02700230	Vaccine Therapy in Treating Patients with Malignant Peripheral Nerve Sheath Tumor That is Recurrent or Cannot Be Removed by Surgery	Recruiting	Oncolytic measles virus encoding thyroidal sodium iodide symporter	I
NCT03872427	Testing Whether Cancers with Specific Mutations Respond Better to Glutaminase Inhibitor, CB-839 HCl, Anti-Cancer Treatment, BeGIN Study	Recruiting	Telaglenastat hydrochloride	II

One of the major hopes for future therapies is using possible gene strategies to restore the malfunction of the *NF1* gene by non-sense suppression and exon skipping to correct neurofibromin deficiency (121, 122). Personalized therapeutic approaches integrating genome editing technology could be used as promising and radical treatments once we fully understand the genotype–phenotype correlations and the important role of modifiers of NF1 (123). However, current studies are not enough to fully understand the genotype–phenotype correlations and heterogeneity of genes in NF1 patients. First, the role of *NF1* mutants and genetic modifiers has not been well-studied in many other clinical features, such as skin-fold freckling or other kinds of cancers. Further clarification is needed to understand the different phenotypes. Moreover, for most of these studies, due to the insufficient size of the cohorts, the link between *NF1* mutants or genetic modifiers of NF1 and phenotypic variabilities remains uncertain. More patients in multicenter studies from different countries are recommended for future research. Moreover, little research has been conducted on Chinese NF1 patients, including basic epidemiological surveys and biomedical and clinical research. Due to the heterogeneity and complexity of *NF1* genotype–phenotype correlations and various genetic modifiers, detailed investigations in the Chinese population are highly needed to provide evidences for evaluating current therapies and provide future personalized medicine for Chinese NF1 patients.

## Author Contributions

WW, C-JW, and X-WC contributed to the conception of the study and wrote the manuscript. Y-HL and Y-HG contributed significantly to the manuscript framework and preparation. BG, Q-FL, and Z-CW help with the writing—review, editing, and supervision. All the authors read and approved the final manuscript.

## Funding

This work was supported by Grants from the Youth Doctor Collaborative Innovation Team Project (QC201803) of Shanghai Ninth People's Hospital, Shanghai Jiaotong University School of Medicine; the Project of Biobank (No. YBKA201901) from Shanghai Ninth People's Hospital, Shanghai Jiao Tong University School of Medicine; the Shanghai Youth Top-Notch Talent Program (201809004), the Chenguang Program supported by Shanghai Education Development Foundation and Shanghai Municipal Education Commission (19CG18), the Science and Technology Commission of Shanghai Municipality (19JC1413), Shanghai Rising-Star Program (20QA1405600), Innovative research team of high-level local universities in Shanghai (SSMU-ZDCX20180700), and Shanghai Municipal Key Clinical Specialty (shslczdzk00901).

## Conflict of Interest

The authors declare that the research was conducted in the absence of any commercial or financial relationships that could be construed as a potential conflict of interest.

## Publisher's Note

All claims expressed in this article are solely those of the authors and do not necessarily represent those of their affiliated organizations, or those of the publisher, the editors and the reviewers. Any product that may be evaluated in this article, or claim that may be made by its manufacturer, is not guaranteed or endorsed by the publisher.

## Abbreviations

NF1: neurofibromatosis type 1
CALMs: cafe-au-lait macules
CNFs: cutaneous neurofibromas
PNFs: plexiform neurofibromas
MPNST: malignant peripheral nerve sheath tumor
SCPs: Schwann cell precursors
ANFs: atypical neurofibromas
ANNUBPs: atypical neurofibromatous neoplasms of uncertain biologic potential
VDR: vitamin D receptor
RNF: ring finger protein
ADCY: adenylate cyclase
OPG: optic glioma.
